# Assessing the relationship between toxicity and economic cost of oncological target agents: A systematic review of clinical trials

**DOI:** 10.1371/journal.pone.0183639

**Published:** 2017-08-22

**Authors:** Francesca Tartari, Alessandro Conti, Roy Cerqueti

**Affiliations:** 1 Department of Economics and Law, University of Macerata. Via Crescimbeni, Macerata, Italy; 2 Azienda Ospedaliera dell’Alto Adige, Bressanone/Brissen Hospital. Via Dante, Bressanone/Brissen, Italy; Universita Campus Bio-Medico di Roma, ITALY

## Abstract

Target agents are peculiar oncological drugs which differ from the traditional therapies in their ability of recognizing specific molecules expressed by tumor cells and microenvironment. Thus, their toxicity is generally lower than that associated to chemotherapy, and they represent nowadays a new standard of care in a number of tumors. This paper deals with the relationship between economic costs and toxicity of target agents. At this aim, a cluster analysis-based exploration of the main features of a large collection of them is carried out, with a specific focus on the variables leading to the identification of their toxicity and related costs. The analysis of the toxicity is based on the Severe Adverse Events (SAE) and Discontinuation (D) rates of each target agent considering data published on PubMed from 1965 to 2016 in the phase II and III studies that have led to the approval of these drugs for cancer patients by US Food and Drug Administration. The construction of the dataset represents a key step of the research, and is grounded on the critical analysis of a wide set of clinical studies. In order to capture different evaluation strategies of the toxicity, clustering is performed according to three different criteria (including Voronoi tessellation). Our procedure allows us to identify 5 different groups of target agents pooled by similar SAE and D rates and, at the same time, 3 groups based on target agents’ costs for 1 month and for the median whole duration of therapy. Results highlight several specific regularities for toxicity and costs. This study present several limitations, being realized starting from clinical trials and not from individual patients’ data. However, a macroscopic perspective suggests that costs are rather heterogeneous, and they do not clearly follow the clustering based on SAE and D rates.

## Introduction

The present study aims at finding out whether there is a clear connection between the toxicity of novel anticancer drugs and their cost. To this end, we explore the information related to the rate of Severe Adverse Events (SAE) and the discontinuation (D) of a qualified set of oncological drugs. Such rates contribute to the creation of a so-called Toxicity Index (TI). Specifically, we have created a high-quality dataset by investigating the phase III studies in the context of the approval by the US Food and Drug Administration (FDA) of the target agents and of their introduction in the clinical practice.

The motivations for our study are of economic and social nature. In fact, cancer is one of the most costly health conditions to manage worldwide [1]. Anticancer agents have represented the 43% of new drugs approved by the FDA in the last decade [2]. The increase of drug spending in oncology is mainly due to the recent introduction of new targeted and immunotherapy agents [3], which have improved the outcome of cancer patients in terms of Overall Survival (OS) and Progression-Free Survival (PFS) compared to conventional chemotherapy. Although these agents are generally associated with a lower rate of treatment D due to drug toxicity, their impact on patients' Quality of Life (QoL) should not be overlooked. Improving patients’ QoL and their compliance to treatments will represent the challenge for cancer researchers in the future years. Indeed, by a purely economic perspective, reducing the toxic effects of these treatments will allow to decrease the abstention from work days and to increase productivity, hence leading to a wider access to cures due to a better economic status [4–8].

This paper can be properly inserted in the frame of pharmacoeconomics, which is a scientific discipline related to the cost and the value of drugs and provides suggestion for the optimal allocation of the healh care resources. This conceptualization was proposed by Townsend in 1987 [9], who identified the Pharmacoeconomics as “the description and the analysis of costs of therapeutic approch substained by the Health System and Society”. However, the first definition of Pharmacoeconomics dates back to 1977 when Weinstein and Stason [10] published a paper dealing with economic analysis in health field.

On the current scenario of rapidly rising health care costs, pharmaeconomic techniques are becoming increasingly relevant to analyze the cost-effectiveness and economic sustainability of emerging drugs [11]. Among such techniques, cluster analysis plays a relevant role. In fact, cluster analysis is used to identify groups of similar data based on selected variables and is particularly suitable for their comparison. The versatility of such a statistical technique explains also its popularity in many fields of applied science [12–20]. Indeed, cluster analysis seems to be appropiate for performing a global study of the connection between drug effectiveness, toxicity and cost. In this context, it is worth mentioning Perrier et al [14], who explored the transferability of health cost assessment between Italy and France. The authors constructed a hierarchical structure using cluster analysis and identified four different clusters based on diagnosis, surgery, chemotherapy and follow-up. Their findings showed that a high variability was present between this two countries, suggesting a low transferability of cost evaluations across Italy and France. Two years later, Liao et al. [15] performed an observational study on 18,380 patients with end-stage renal disease who initiated hemodialysis. By using K-means and hierarchical cluster analyses with either flexible beta or Ward’s methods, they identified 4 clusters based on sample sizes and change of cost patterns, finding that higher costs were correlated with more increasing comorbidity scores.

In our study we are different from the quoted papers since we first create a dataset containing clinical and economic information about all the oncological target agents approved in clinical practice. In this respect, it is important to recall that a target agent is a drug that is able to recognize one or more specific molecules expressed by tumor cells, immune cells or, more generally, by tumor microenvironment in cancer patients. The identification procedure has been rather complex -it mirrors the complexity of the faced problem- and represents a relevant step of the research.

Furthermore, we have employed a method based on Voronoi tessellation [21], which represents a potential visualization of the subgroups identified by the cluster analysis. Voronoi diagram is a kind of decomposition of a given metric space based on the distance (which is Euclidean in the original formulation of Voronoi) to specified sites called centroids [21]. Particularly, each centroid recognizes data that are nearer to it than to the other centroids in accord to the given distance. By applying this technique, we are able to explore the way in which clusters of toxicity and costs overlap, hence giving information on the relationship between drug toxicity and related cost.

As we will see below, to gain more insights we depart from the original formulation of Voronoi and consider also minimum and maximum distances.

Cluster analysis, with a specific Voronoi diagrams approach, has been recently applied in the economic field [22–25]. In 2009, Liu et al. [22] explored the distribution of rural assessment using this technique. They showed that the distance from highways and rivers were the two factors that majorly influenced the distribution of rural settlements. More recently, Vaz et al. [23] reported a significant difference in term of regional innovation patterns as a consequence of istitutional innovation profiles.

As already mentioned above, we here investigate, through a cluster analysis procedure, whether there is a correlation between the cost of molecularly targeted and immnotherapy agents and their toxicity in terms of SAE and D rates.

To the best of our knowledge, this is the first paper dealing with toxicity and cost of target agents in oncology through a cluster analysis. More than this, the construction of the dataset on the basis of an exhaustive literature review is also a novelty in the oncological studies.

The rest of the paper is organized as follows. Section 2 collects the results of the analysis, while Section 3 provides a discussion of them. In Section 4 we present how the used dataset has been constructed and illustrate its main statistical properties. Furthermore, Section 4 contains also the description of the employed methodological tools, with a detailed explanation of the cluster analysis.

## Materials and methods

### Costruction of the dataset

The construction of the dataset has been implemented through a critical analysis of a wide set of clinical studies.

The selection of the relevant researchs has been carried out according to the instructions contained in the PRISMA [26] (S1 File). The scientific literature of interest has been identified from keywords selections on the PubMed database, in a period ranging from 1965 to 2016. Specifically, the research has been conducted by combining the words "cancer", "neoplasm", "solid tumor" and "clinical trial" with the name of each target agent.

As a second step, we have identified the papers dealing with human studies and randomized trials published in English and meeting the following criteria: 1) phase III studies conducted in patients with cancer; 2) random assignment of participants to treatment with a target therapy or a control (standard of care, placebo or best supportive care). In case of several publications related to the same experiment, only the most recent one or the most complete referring to included trial has been considered. Phase I and phase II trials has been excluded because of their variability and the lack of sufficient controls.

For each of the obtained papers we have reported the scientific study, the name of all authors, the name of the journal, the reference year, the number of the volume and the reference pages.

The resulting list of studies on target agents has been explored to assess the variables of interest related to the specific agent, i.e.: number of patients treated with target agents in the clinical studies, PFS (defined as the time from the start of therapy to disease progression or death), rate of all-grade AE and SAE (which leads to the necessity of medical assistence, hospitalization or drug interruption) and the D rate due to drug toxicity.

For the present research, we consider as variables the rate of SAE and the D rate, leaving the other ones for future studies.

Information on the costs of the target agents has been derived directly from their websites. All costs are expressed in American US Dollars.

### Cluster analysis

Cluster analysis and Voronoi tesselation were performed by R software version 3.3.0 for Windows (62 megabytes, 32/64 bit). We have compared the clusters of target agents obtained when taking toxicity and when taking costs.

For what concerns toxicity, we have considered SAE and D rates as relevant variables. They are the parameters concurring in our conceptualization of the *Toxicity Index* (*TI*, hereafter).

The procedure of centroids selection has been implemented accordingly to clinical and scientific criteria, in order to represent the most meaningful groups of combinations of the two variables. For this analysis, we have reasonably considered five centroids as follows: *ϕ*_1_ = (10,5); *ϕ*_2_ = (30,15); *ϕ*_3_ = (45,10); *ϕ*_4_ = (60,20); *ϕ*_5_ = (75,25), where the first component is the SAE value while the second one represents the D rate. In particular, centroid *ϕ*_1_ is associated with low rate of SAE and low D rate, which leads to a low TI; *ϕ*_2_ has low-medium rate of SAE and medium D rate, which means low-medium TI; *ϕ*_3_ has medium rate of SAE and low-medium D rate (medium TI); *ϕ*_4_ represents medium-high rate of SAE and medium-high D rate (medium-high TI); *ϕ*_5_ identifies a cluster with high rate of SAE and high D rate (high TI). The cluster obtained by centroid *ϕ*_*h*_ will be denoted by *C*_*h*_, for each *h* = 1,2,3,4,5. Moreover, by denoting the observations of SAE and D rates by the variables *x* and *y*, respectively, we also denote components of the centroid *ϕ*_*h*_ = (*ϕ*_*h*,*x*_,*ϕ*_*h*,*y*_), for each *h* = 1,2,3,4,5.

Clusters are identified by the nearness of the target agent toxicity with the centroids. At this aim, we apply three different concepts of distance: an Euclidean one–in accord to the original model of Voronoi-, the maximum and the minimum. Formally, for any given target agent *j* = 1,2,…,37 with SAE rate *x*_*j*_ and D rate *y*_*j*_, we define

$$d_{E}(j,\phi_{h})=\sqrt{{\left(x_{j}-\phi_{h,x}\right)}^{2}+{\left(y_{j}-\phi_{h,y}\right)}^{2}}$$

$$d_{M}(j,\phi_{h})=max\{{|x}_{j}-\phi_{h,x}|,|y_{j}-\phi_{h,y}|\}$$

$$d_{m}(j,\phi_{h})=min\{{|x}_{j}-\phi_{h,x}|,|y_{j}-\phi_{h,y}|\}$$

According to the specific metric selected, we derive the clusters of target agents as follows:

$$C_{h}^{K}=\{j=1,\ldots ,37|\;d_{K}(j,\phi_{h})<d_{K}(j,\phi_{\bar{h}}),\forall \bar{h}\neq h\},\;\forall \;K=E,M,m,\;\forall \;h=1,2,3,4,5.$$

For what concerns the costs of the target agents, we have implemented two simple clusterings based on two variables. First, we have grouped the investigated drugs into three groups on the basis of 1-month cost patterns: cost less than 7,000$ (Group A), cost ranging from 7,000 to 11,000$ (Group B) and cost greater than 11,000$ (Group C). In the same way, drugs were grouped according to their costs extimated for the complete treatment for each patient within 3 groups: cost less than 40,000$ (Group D), cost ranging from 40,000$ to 80,000$ (Group E) and cost greater than 80,000$ (Group F).

## Results

At the end of text analysis, we have obtained 4,803 studies concerning the use of molecular targeted drugs in cancer patients (the list of drugs is reported in the first column of Table 1).

**Table 1:** List of target agents employed in oncological patients. Their characteristics are related to drug efficacy in terms of median Progression-Free Survival (PFS) and drug toxicity in terms of rate of all-grade, severe adverse events and discontinuation rate. BCC = Basal-cell Carcinoma; GIST = Gastrointestinal Stromal Tumor; NSCLC = Non Small Cell Lung Cancer; RCC = Renal Cell Carcinoma.

Target Agent	First Authors, Year	Reference	Cancer Type	Number of Patients	Median PFS (Months)	All grade Adverse Events (%)	Severe Adverse Events(%)	D Rate (%)
Abiraterone acetate (first line therapy)	Charles JR, 2013	27	Prostate	546	16.5	99	48	10
Abiraterone acetate (successive line-therapy)	de Bono S, 2011	28	Prostate	797	5.6	23	7	19
Afatinib	Sequist LV, 2013	29	NSCLC	230	11.1	NA	49	8
Bevacizumab	Friedman HS, 2009	30	Glioblastoma	82	5.6	100	65.8	17.7
Bevacizumab	Escudier B, 2007	31	RCC	327	10.2	97	29	28
Bevacizumab (first line therapy)	Hurwitz H, 2004	32	Colorectal	411	10.6	NA	84,9	8.4
Bevacizumab (successive line-therapy)	Bennouna J, 2013	33	Colorectal	409	5.7	98	64	16
Cabozantinib	Eisei R, 2013	34	Thyroid	219	11.2	NA	69	16
Cetuximab	Vermorken JB, 2008	35	Head and Neck	222	5.5	NA	82	20
Cobimetinib + Vemurafenib	Larkin J, 2014	36	Melanoma	247	9.9	95	62	12
Crizotinib	Shaw AT, 2013	37	NSCLC	173	7.7	NA	33	6
Enzalutamide (first line therapy)	Beer TM, 2015	38	Prostate	800	8.3	34	28	8
Enzalutamide (successive line-therapy)	Scher HI, 2012	39	Prostate	872	5.7	97	43	6
Erlotinib	Moore MJ, 2007	40	Pancreas	282	3.8	100	61	10
Erlotinib (first line therapy)	Rosell R, 2012	41	NSCLC	86	9.7	98	45	13
Erlotinib (maintainance therapy)	Cappuzzo F, 2010	42	NSCLC	438	2.9	NA	11	16
Everolimus	Baselga J, 2012	43	Breast	482	7.8	NA	23	19
Lenvatinib	Schlumberger M, 2015	44	Thyroid	261	14.7	97.3	75.9	14.2
Nivolumab	Brahmer J, 2015	45	Squamous NSCLC	135	3.5	58	7	3
Nivolumab	Borghaei H, 2015	46	Non-Squamous NSCLC	292	2.3	69	10	5
Nivolumab	Robert C, 2015	47	Melanoma	210	5.1	74.3	11.7	2.4
Nivolumab	Motzer RJ, 2015	48	RCC	410	4.6	79	19	8
Palbociclib (+letrozole)	Finn RS, 2015	49	Breast	84	20.2	99	76	33
Palbociclib (+fulvestrant)	Turner NC, 2015	50	Breast	347	9.2	97.7	69,3	2.6
Pembrolizumab	Robert C, 2015	51	Melanoma	277	4.1	72.9	75	6.9
Ramucirumab	Fuchs CS, 2014	52	Gastric	238	2.1	94	57	11
Ramucirumab	Garon EB, 2014	53	NSCLC	628	4.5	98	79	15
Ramucirumab	Tabernero J, 2015	54	Colorectal	536	5.7	83	36	11
Regorafenib	Grothey A, 2013	55	Colorectal	505	1.9	93	54	44.8
Sonidegib	Midgen MR, 2015	56	BCC	79	13.1	95	31	22
Sorafenib	Escudier B, 2007	57	RCC	451	5.5	NA	34	10
Sunitinib	Motzer RJ, 2009	58	RCC	375	11	NA	7	38
Sunitinib	Demetri GD, 2006	59	GIST	207	6.4	83	20	9
T-DM1	Verma S, 2012	60	Breast	495	9.6	95.9	15,5	5
Temsirolimus	Hudes G, 2007	61	RCC	209	3.8	NA	11	7
Trametinib + Dabrafenib	Long GV, 2014	62	Melanoma	211	9.3	95	32	9
Ziv-Aflibercept	Van Cutsem E, 2012	63	Colorectal	612	6.9	99.2	83,5	26.8

Therefore, 2,914 of the 4,083 original papers have been excluded because of phase I studies, observational, in vitro, reviews or letters about targeted therapies. Of the 1,889 remained studies, 1,852 were excluded because dealing with phase II or because not containing data on the SAE and D rates.

As a result, we have found 23 target agents that are used in 37 different therapeutic settings [27–63] (Table 1).

**Fig 1:**
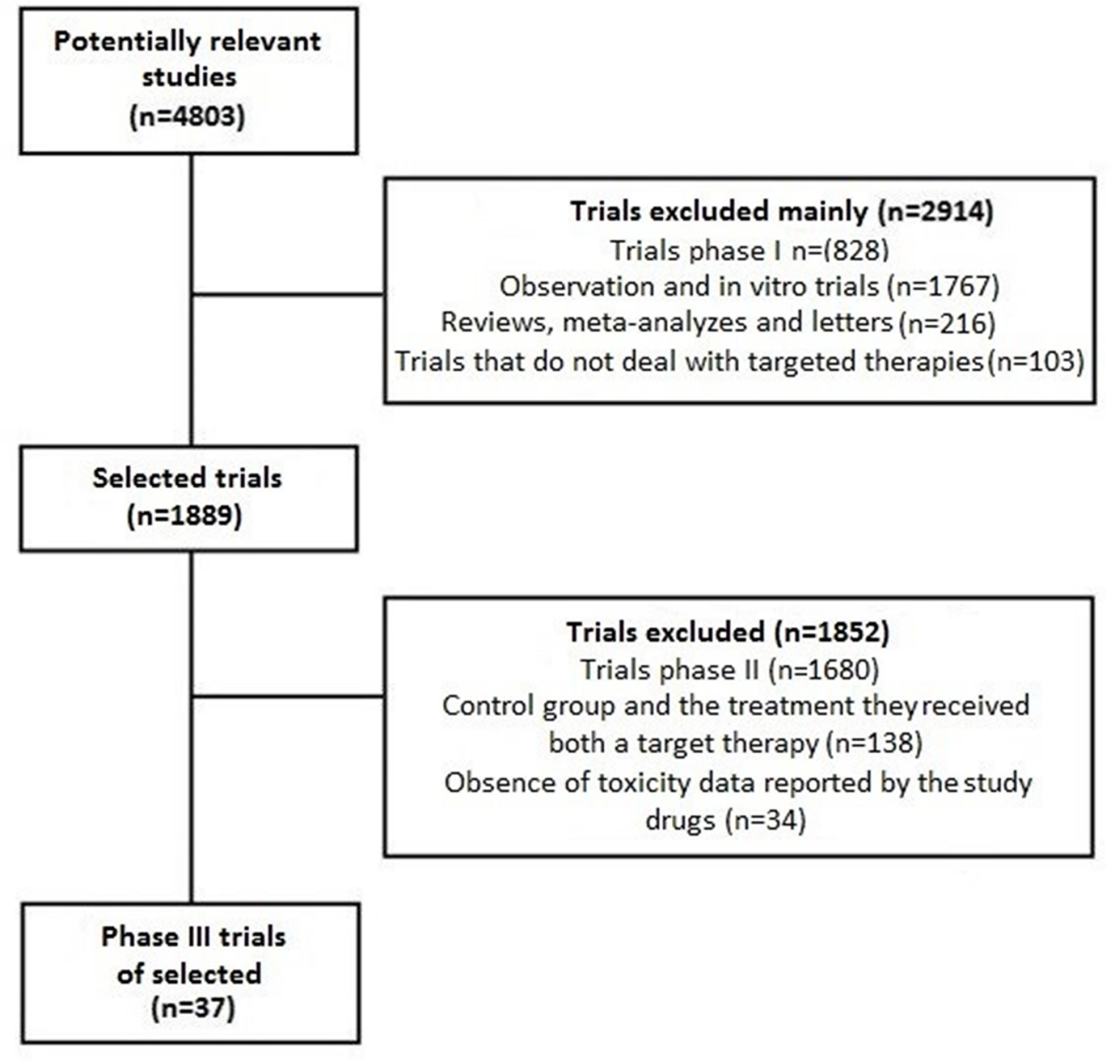
Study selection according to PRISMA statement.

The identification of the relevant papers is described in Fig 1, where it is presented a block diagram of the PRISMA procedure.

Table 2 contains the main statistical indicators of the dataset. The mean/std. dev. ratio allows additional considerations about the heterogeneity within the clusters, which is low, supporting that each cluster includes similar drugs both in terms of SAE and D rates.

**Table 2:** Main statistical indicators of the dataset.

	Number of patients	DRUG EFFECTIVENESS	DRUG TOXICITY		
		Median PFS (months)	All grade adverse events (%)	Severe adverse events (%)	Discontinuation rate (%)
Mean	356	7.60	86	44	14
Std. Dev.	205	4.14	20	26	10
Mean/Std. Dev.	1.73	1.84	4.30	1.68	1.42
Min	79	1.9	23	7	2.4
Max	872	20.2	100	84.9	45
Median	292	6.4	95	43	11
Skewness	0.83	1.03	-2.06	0.10	1.48
Kurtosis	0.23	1.21	3.95	-1.38	2.19
Q1	211	4.6	81	20	8
Q3	482	9.9	98	65.8	17.7

Concerning skewness, it is relevant to note that only the rate of all grade adverse events is negative (-2.06) with a curve of distribution characterized by a longer left tail with a median of patients developing at least an adverse event (95%) that overcross the mean of patients (86%). Further information can be added by observing the leptokurtic distribution of all grade adverse events (curtosis is 3.95), while the distribution of SAE is platykurtic (curtosis is -1.38).

It is also important to observe the response rates reported by target agents, which range from 1% to 80% (Table 2). Such a result underlines the extreme variety of actions of these new generation agents that can improve patient survival without reducing tumour sizes.

**Fig 2:**
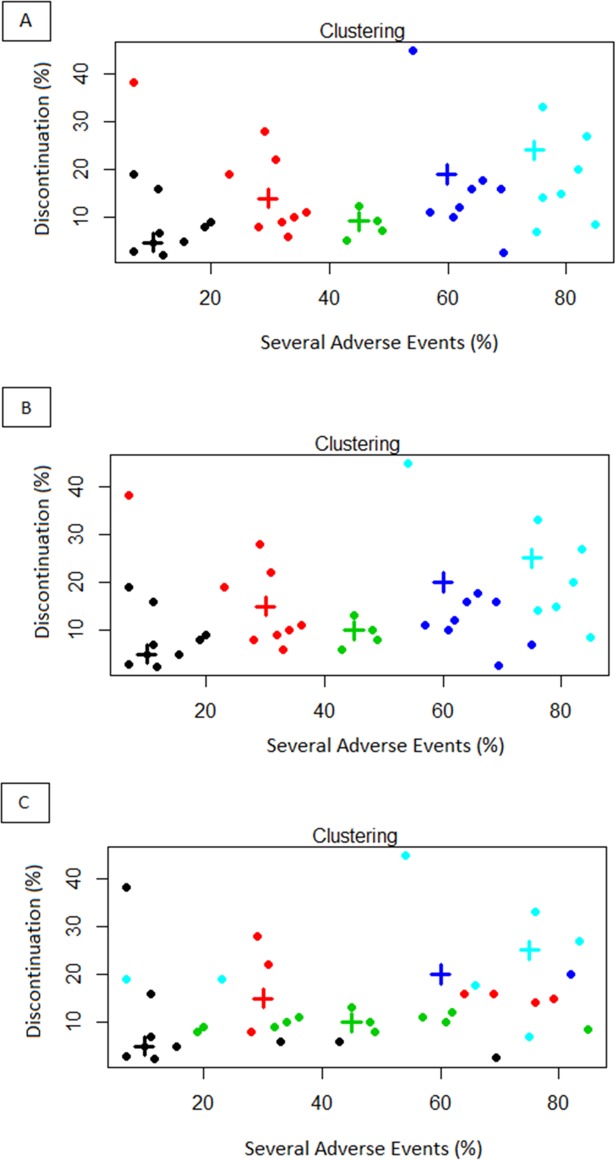
Cluster analysis based on Toxicity Index (TI) considering Euclidean distance (A), maximum distance (B) and minimum distance (C). The “+” represent the centroids.

**Fig 3:**
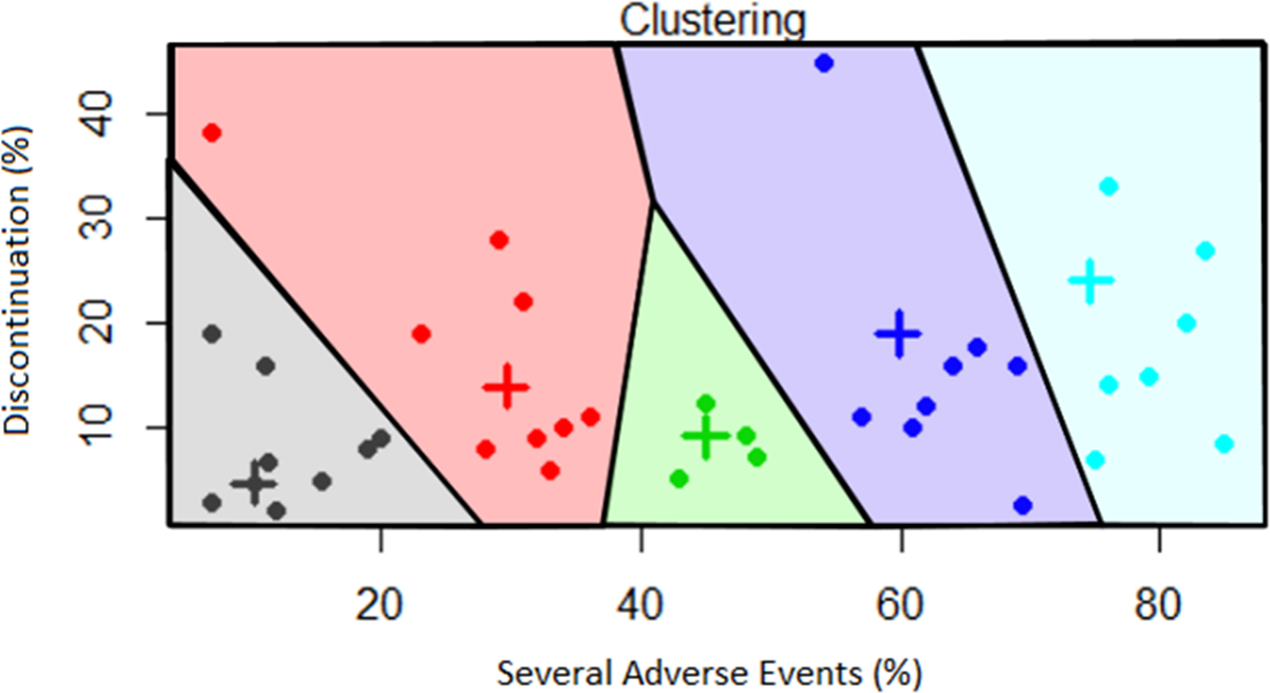
Voronoi tesselation based on Toxicity Index (TI) considering Euclidean distance.

Fig 2*A*, 2*B* and 2*C* show the clusters based on Euclidean distance, maximum distance and minimum distance, respectively. A spatial representation of the dynamic fields related to cluster analysis by Euclidean distance has been obtained by Voronoi diagram as reported in Fig 3.

The results of cluster analysis with Euclidean distance show a major similarity with the findings obtained by the maximum distance. In particular, such clustering criteria place in two different clusters only two drugs (Regorafenib, charaterized by SAE and D rates of 54 and 44.8, respectively, and Pembrolizumab, with SAE and D rates of 75 and 6.9, respectively. They belong to cluster 4 based on Euclidean distance and to cluster 5 according to the maximum distance). Differently, the clusters based on minimum distance are markedly different from both the other analyses.

It is interesting to note that the highest cost for a month and per PFS are represented by the combination of Cobimetinib and Vemurafenib and the lowest by Erlotinib (when used for patients with pancreatic cancer). The mean and median montly costs are 9,366 $ and 8,627 $, respectively. On the other hand, the mean and median costs per PFS are 73,154 $ and 49,500 $, respectively (Table 3).

**Table 3:** List of target agents approved for their use in cancer patients and related costs. BCC = Basal-cell Carcinoma; GIST = Gastrointestinal Stromal Tumor; NSCLC = Non Small Cell Lung Cancer; RCC = Renal Cell Carcinoma.

Target Agent	Cancer Type	Monthly cost ($)	Cost per PFS ($)
Abiraterone acetate (first line therapy)	Prostate	8,627	142,346
Abiraterone acetate (successive line-therapy)	Prostate	8,627	48,311
Afatinib	NSCLC	6,970	77,367
Bevacizumab	Glioblastoma	4,400	24,640
Bevacizumab	RCC	4,400	44,880
Bevacizumab (first line therapy)	Colorectal	2,680	28,408
Bevacizumab (successive line-therapy)	Colorectal	2,680	15,276
Cabozantinib	Thyroid	14,300	160,160
Cetuximab	Head and Neck	7,000	38,500
Cobimetinib + Vemurafenib	Melanoma	26,300	260,370
Crizotinib	NSCLC	11,500	88,550
Enzalutamide (first line therapy)	Prostate	7,450	61,835
Enzalutamide (successive line-therapy)	Prostate	7,450	42,465
Erlotinib	Pancreas	2,450	9,310
Erlotinib (first line thrapy)	NSCLC	3,000	29,100
Erlotinib (maintainance therapy)	NSCLC	3,000	8,700
Everolimus	Breast	7,000	54,600
Lenvatinib	Thyroid	13,945	204,992
Nivolumab	Squamous NSCLC	12,600	44,100
Nivolumab	Non-Squamous NSCLC	12,600	28,980
Nivolumab	Melanoma	12,600	64,260
Nivolumab	RCC	6,984	32,126
Palbociclib (+letrozole)	Breast	9,850	198,970
Palbociclib (+fulvestrant)	Breast	9,850	90,620
Pembrolizumab	Melanoma	23,017	94,370
Ramucirumab	Gastric	13,000	27,300
Ramucirumab	NSCLC	11,000	49,500
Ramucirumab	Colorectal	13,000	74,100
Regorafenib	Colorectal	7,600	14,440
Sonidegib	BCC	12,000	157,200
Sorafenib	RCC	6,600	36,300
Sunitinib	RCC	7,000	77,000
Sunitinib	GIST	7,000	44,800
T-DM1	Breast	9,800	94,080
Temsirolimus	RCC	2,960	11,248
Trametinib + Dabrafenib	Melanoma	16,300	151,590
Ziv-Aflibercept	Colorectal	11,000	75,900

**Fig 4:**
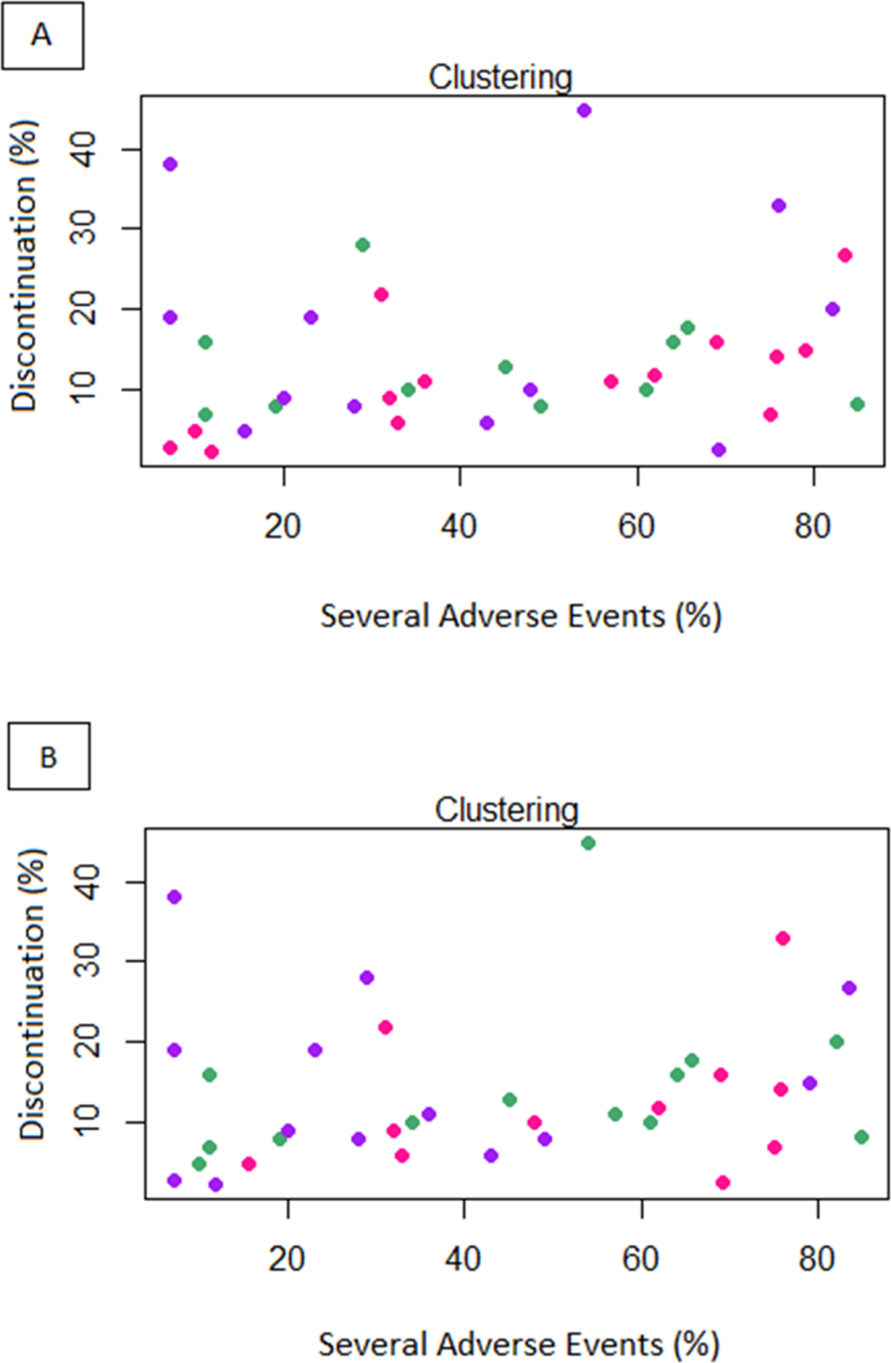
Cluster analysis based on Euclidean distance considering the drug costs for 1-month (A) or for the median total duration of therapy (B) for a single oncological patient. Green points represent drugs with low cost (Group A), violet points drugs with medium cost (Group B) and pink with high cost for 1-month of treatment (Group C).

**Fig 5:**
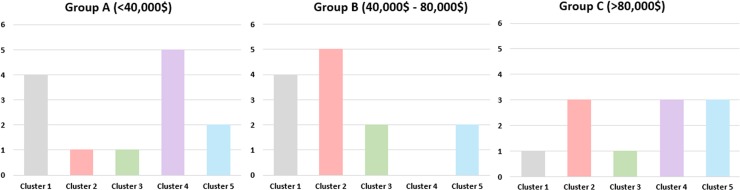
The distribution of different clusters into the three cost categories related to the amount for median Progression-Free Survival (PFS).

Both Fig 4*A* and 4*B* show that heterogeneous cost distribution that doesn’t clearly follow the cluster division based on TI. However, some illustrations of the relationship between toxicity and costs can be carried out at the single clusters level. For instance, as for the 1-month cost, the higher rate of drugs from Group C belongs to Cluster 5, whilst the higher percentage of drugs from Group A are included in Cluster 3. Concerning the total cost estimated for a single patient for the whole treatment, the higher rate of drugs belonging to Group D belongs to Cluster 4, whilst the higher percentage of drugs from Group F are in Cluster 5. The complete distribution of costs within the 5 clusters is reported in Table 4 and Fig 5.

**Table 4:** Distribution of costs within the 5 clusters based on TI.

	1-month treatment cost			Total cost for a single patient (estimated by PFS)		
	*Group A (%)*	*Group B (%)*	*Group C (%)*	*Group D (%)*	*Group E (%)*	*Group F (%)*
*Cluster 1*	33	33	34	44	44	12
*Cluster 2*	22	34	44	11	56	33
*Cluster 3*	50	50	0	25	50	25
*Cluster 4*	38	24	38	63	0	37
*Cluster 5*	14	28	58	29	29	42

## Discussion

Our paper concerns the study of the relationship between the toxicity and cost of newly approved target agents in the Oncology field. All the drugs approved by FDA have been considered. Variables related to SAE and D rates have been collected from published phase III studies.

Cluster analysis has been employed to explore such a relationship. Specifically, three different clustering criteria based on the Euclidean distance–in accord to the standard Voronoi tessellation definition- and maximum and minimum distances have been considered.

To interpret the outcomes of the analysis, we need to provide an intuitie description of the clustering criteria.

The minimum distance is the one that underestimate the toxicity level, in that it may place a drug in a low-toxicity cluster even if some related parameters are of remarkable high level.

Differently, the maximum distance is more “cautios” and overestimates the level of toxicity, since it may insert an agent into a high-toxicity cluster even when some toxicity parameters exhibit a low value.

The “fair” situation is captured by the Euclidean distance, which is the one used in the original Voronoi model. The comparison among the results of the clustering proedures suggests that taking a definition of toxicity that may imply its overestimation is closer to fairness than dealing with an understimation criterion.

It is important to note that the toxicity associated with oncological drugs implicates a high-cost management. In this regard, previous studies have tried to quantify this amount. For example, Roncato et al. [64] evaluated the economic burden of Irinotecan-related toxicity in patients with metastatic colorectal cancer, revealing that the mean predicted cost per patient was 4,886 €. On the other hand, Arondekar et al. [65] investigated the costs of AEs in 2,621 patients with metastatic melanoma by employing multivariate generalized linear models (GLMs) with a log-link function and gamma distribution. They reported a 30-day incremental cost of over 9,000 $ for metabolic AEs, 8,450 $ for hematologic, 6,476 $ for cardiovascular and 6,338 $ for gastrointestinal AEs [65].

Similarly, Bilir et al. [66] studied the economic burden of toxicities associated with treating metastatic melanoma in the United States. They registered that the highest mean in patient costs for an AE were associated with acute myocardial infarction, sepsis, and coma, ranging from 31,682 $ to 47,069 $. In addition, the mean cost for hospitalization due to other AEs ranged from 19,122 $ to 26,861 $ [66].

The quantification of the economic impact related to the toxicity of target agents will represent a major step forward in the phases of drug approval and cost establishement, representing a fundamental parameter that must be considered during these processes.

In the last years, several techniques of drug reimboursement have been introduced in the pharmacoeconomic scenario and are currently employed in the oncological field. These modalities include: (1) payment by results, which consists in the total refund by the manyfacturer for non-responding patients; (2) risk sharing, which provides for a partial refund for non-responding patients after a clinical/radiological evalutation; (3) cost sharing, which sets an initial discount for all treated patients. These techniques have become even more fundamental after the introduction of immunotherapy in the therapeutic armamentarium of cancer patients. In fact, these agents are characterized by both relevant cost and high efficacy, supporting the research for new tools aimed to optimize the use of economic resources in the health system. In this respect, Russi et al. [67] proposed a new cost-containmet strategy for the use of immunotherapic agent ipilimumab for patients with melanoma in Italy. This model included, by one side, drug-day and centralization of compounding (accounting for a reduction of -11.1% of drug cost) and, by the other side, payback systems designed by AIFA (resulting in additional -6.2%) [67].

Our study present several limitations. First of all it is a systematic review realized starting from clinical trials and not from individual patients’ data. Thus, data on drug toxicity might be influenced by confounding factors such as the presence of different tumors, patients’ comorbidities or simultaneous treatments. Furthermore, patients eligible for clinical trials mostly show fair organ functions, leading to a potential underestimation of drug toxicity compared to clinical practice. Finally, we are awared that the various toxicities considered in our analysis may have a different impact on patient QoL and a wide range of clinical consequences, although we considered only SAEs that lead to patient hospitalization and/or medical interventions and the D rate.

In face of these limitations, at a macroscopic level, our analysis highlights that there is a not straightforward relationship between the toxicity of target agents and their relative costs for 1-month or the whole treatment duration. However, we can notice that the number of target agents with high costs results more relevant in the clusters associated with the worst drug-tolerability (high SAE and D rates), although they belong also to the cluster characterized by better safety (low SAE and D rates).

Interestingly, data on kurtosis and skewness underline that a high percentage of cancer patients treated with molecularly target agents do experience at least one all grade adverse event. The toxicity of these drugs, altough lower than that associated with chemotherapy, suggest that the costs of management of adverse events must be considered during the phases of approval and price negotiation.

To sum up, the relationship between the cost and the efficacy and toxicity of new generation drugs does not follows a regular path. However, the constructed database and the findings here obtained can be efficiently used for the development of a unified theory on the cost management of treating cancer patients and on the study of the impact of these agents on their QoL.

## Supporting information

S1 File PRISMA 2009 checklist. List of the indications provided by the “PRISMA statment” for the realization of meta-analyses and systematic review.
(DOC)
